# Supplementary figures and images for: Virtual tissue microstructure reconstruction across species using generative deep learning
Source: PLoS One. 2024 Jul 12;19(7):e0306073. doi: 10.1371/journal.pone.0306073 (PMC11244806; doi:10.1371/journal.pone.0306073)

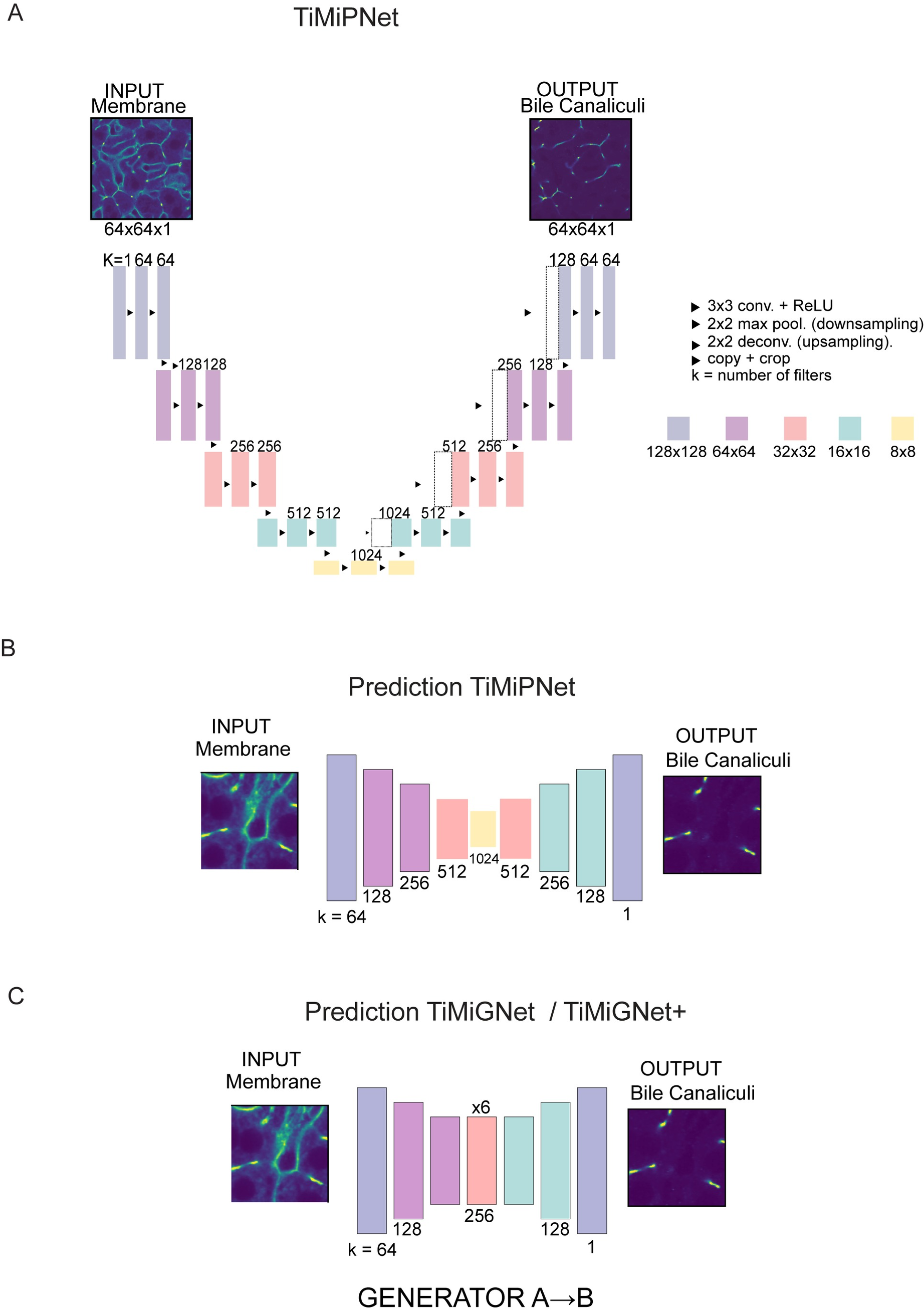

Supplement: S1 Fig — a) Schematic representation of the training framework for TiMiPNet. b) Schematic representation of the prediction framework for TiMiPNet. c) Schematic representation of the prediction framework for TiMiGNet and TiMiGNet+. (TIF) [file pone.0306073.s001.tif]

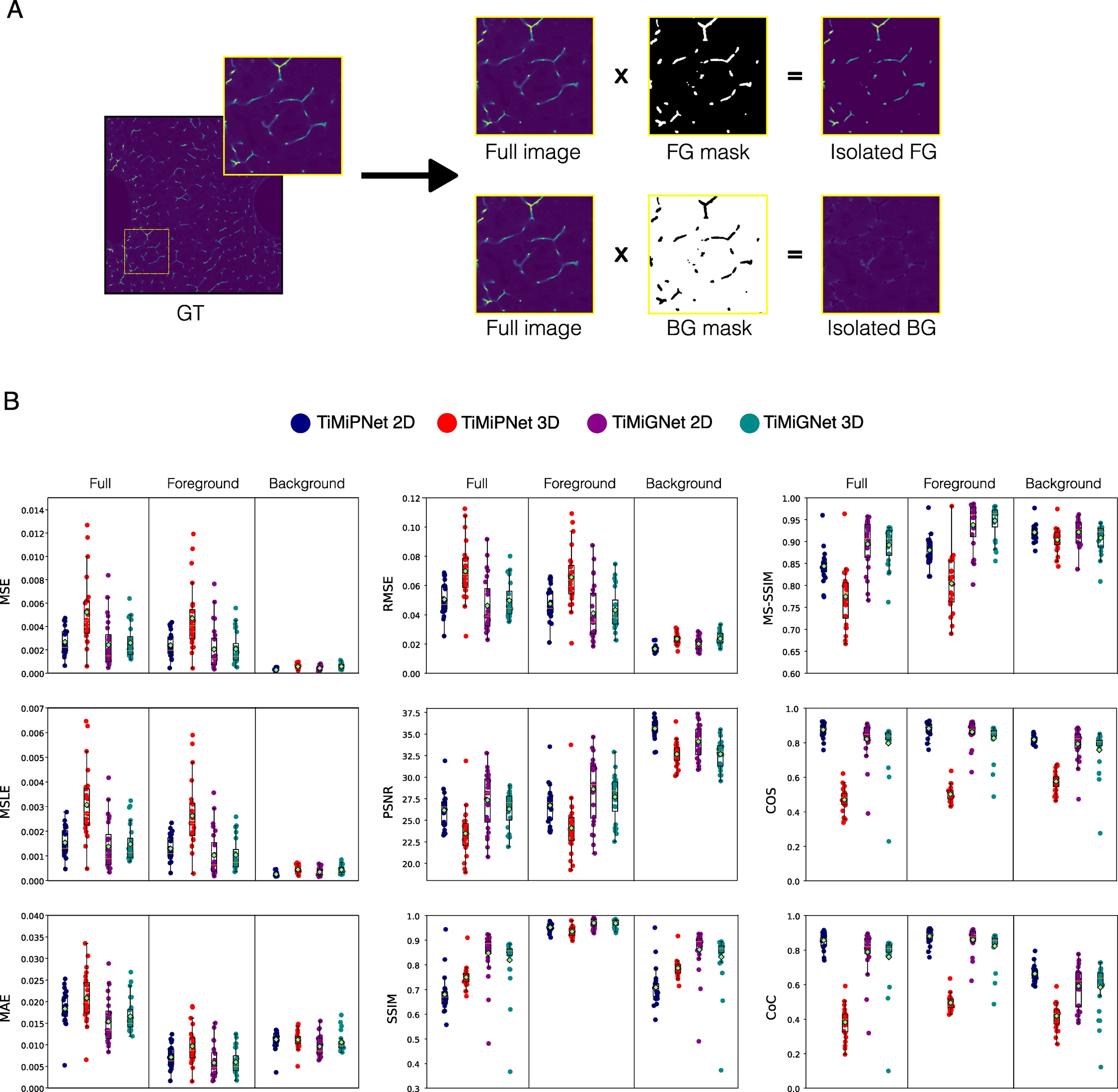

Supplement: S2 Fig — a) Schematic representation of the process for generating the mask to split background from foreground in ground truth samples. The masks were calculated using the Otsu method for binary thresholding. b) Quantification of the performance of the predictions of the BC network generated by the 2D and 3D, TiMiPNet, and TiMiGNet models using the following metrics Mean Squared Error, Mean Squared Logarithmic Error, Mean Absolute Error, Root Mean Squared Error, Peak Signal-to-Noise Ratio, Structural Similarity Index Measure, Multi-scale Structural Similarity Index Measure, Cosine Similarity, and Coefficient of Correlation. The test images were splitted in 128x128x128 cubes and the metrics were estimated independently for each cube, i.e. each dot represents one image cube. The box plots enclose values from the lower to upper quartiles. The middle line represents the median and the whiskers show the data range. (TIF) [file pone.0306073.s002.tif]

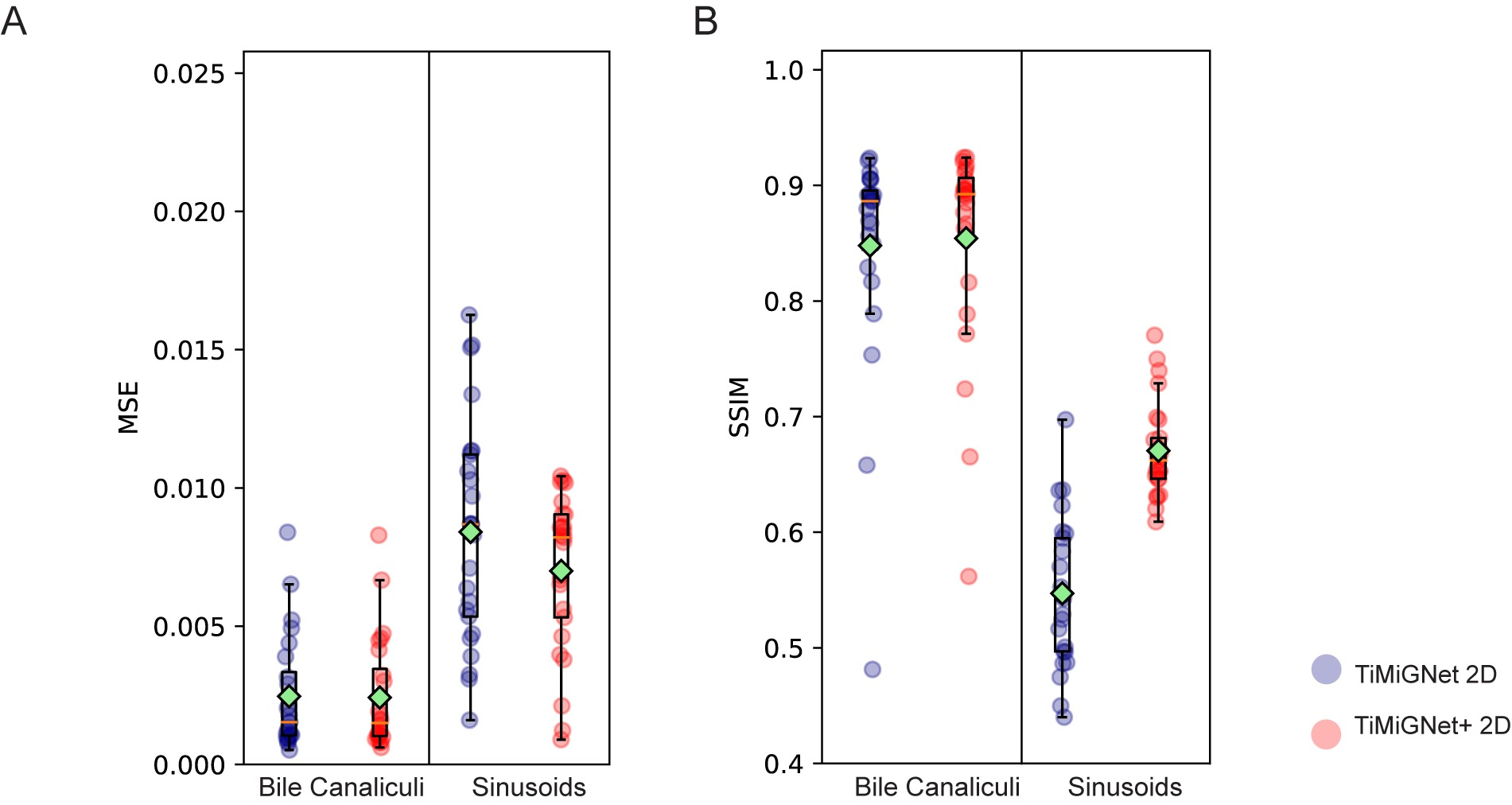

Supplement: S3 Fig — Quantification of a) Mean Squared Error and b) Structural Similarity Index Measure for the predictions generated by TiMiGNet and TiMiGNet+ when compared with the experimental images. The test images were splitted in 128x128x128 cubes and the metrics were estimated independently for each cube, i.e. each dot represents one image cube. The box plots enclose values from the lower to upper quartiles. The middle line represents the median and the whiskers show the data range. (TIF) [file pone.0306073.s003.tif]

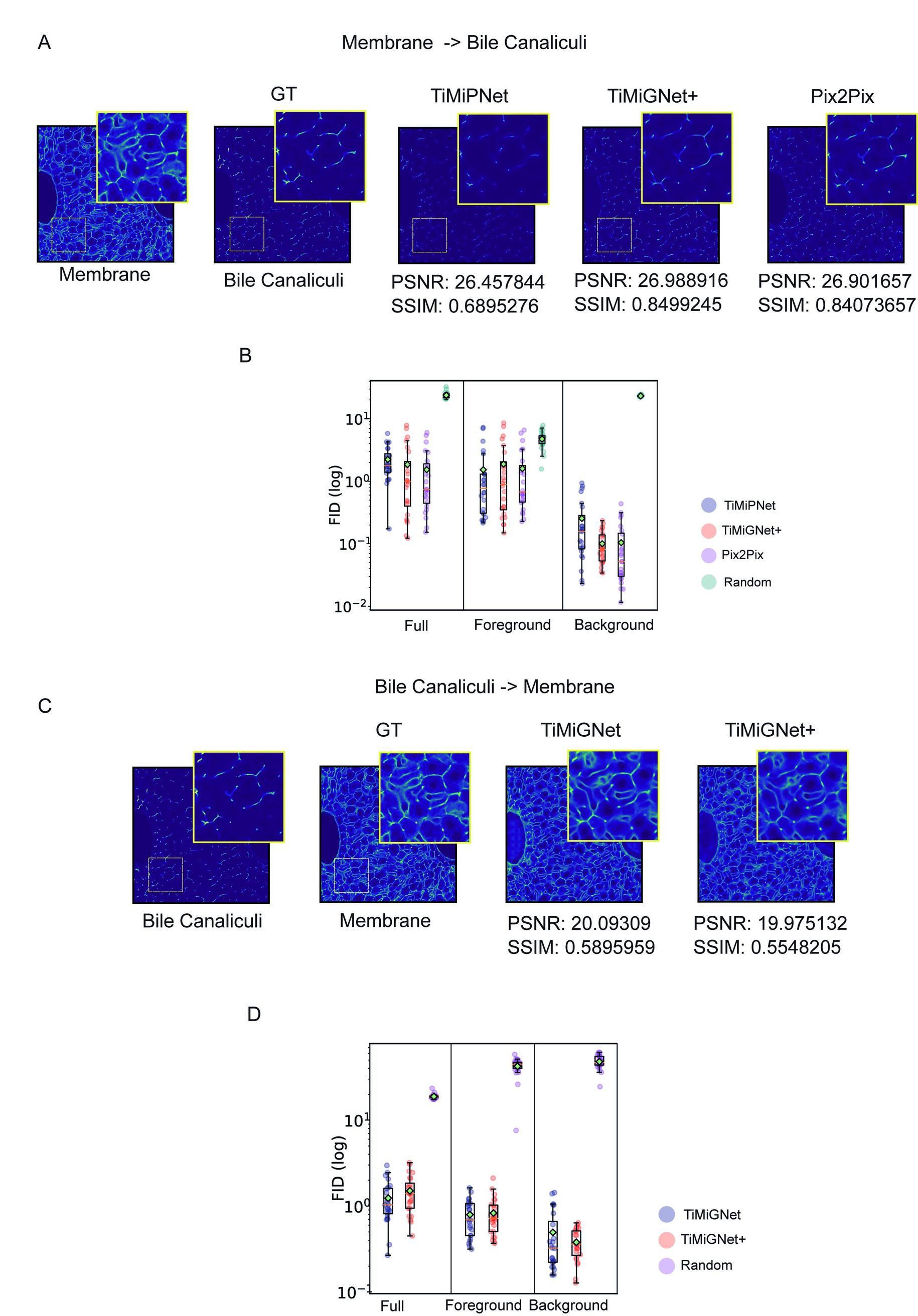

Supplement: S4 Fig — (a) 2D sections of 3D fluorescent images of the actin mesh (membranes) and Bile Canaliculi (experimental images) as well as the corresponding predictions by TiMiPNet, TiMiGNet+ and Pix2Pix in 2D. b) Quantification of the performance of the predictions of the BC network generated by the models. c) 2D sections of 3D fluorescent images of the BC and actin mesh as well as the corresponding predictions of the generators of TiMiPNet and TiMiGNet+. b) Quantification of the performance of the predictions of the BC network generated by the models. (TIF) [file pone.0306073.s004.tif]

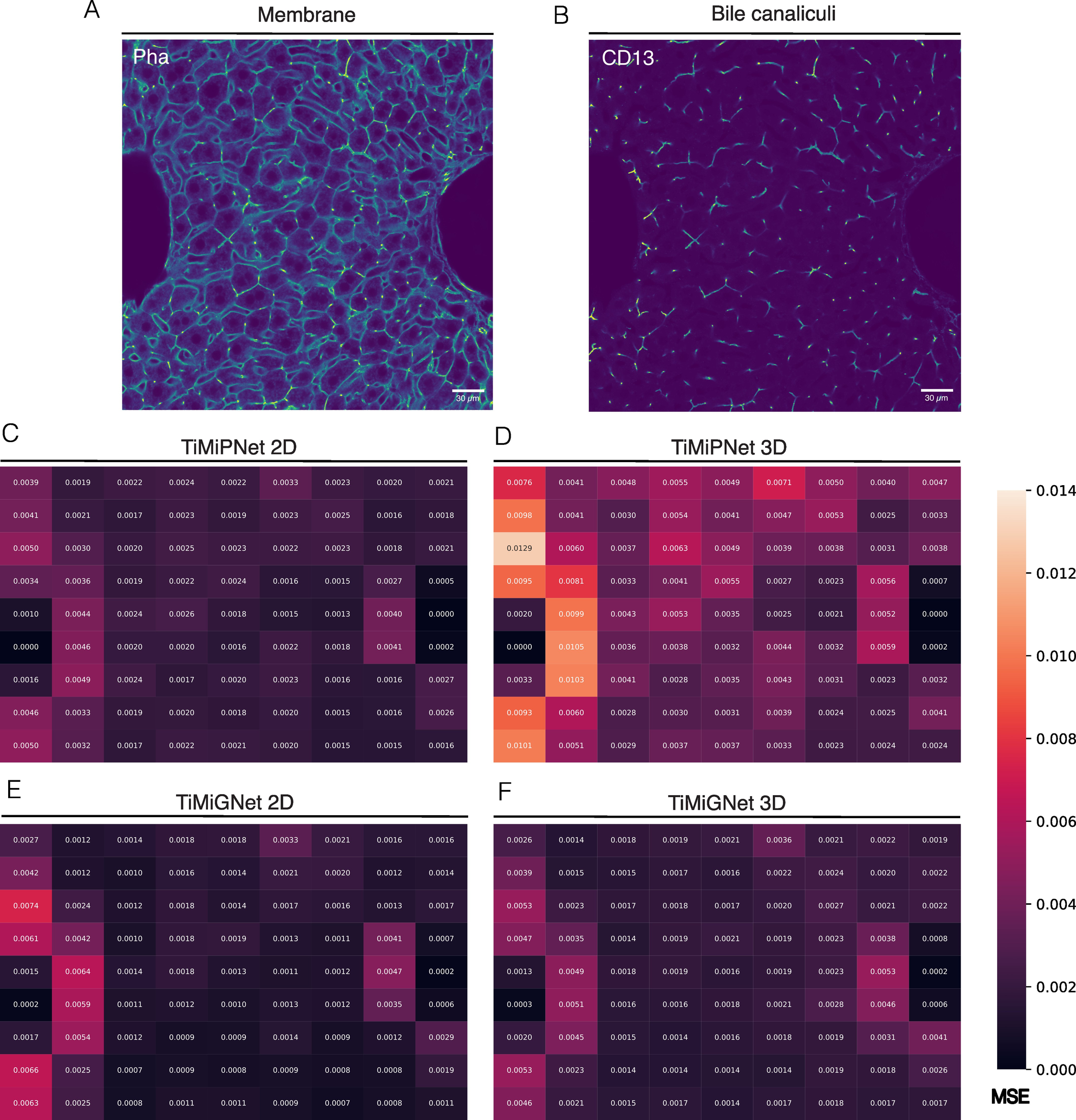

Supplement: S5 Fig — (a-b) Representative images actin mesh (membranes), Bile Canaliculi. (c-f) the images were divided into 9x9 blocks and the mean squared error of the predictions of the different models was calculated and shown as a heat map. Whereas low values show good agreement with the ground truth, high vales show potential mispredictions. (TIF) [file pone.0306073.s005.tif]
