# Supplementary material for: Virtual tissue microstructure reconstruction across species using generative deep learning
Source: PLoS One. 2024 Jul 12;19(7):e0306073. doi: 10.1371/journal.pone.0306073 (PMC11244806; doi:10.1371/journal.pone.0306073)
Supplement: S1 Table — Average PSNR, SSIM and MSE on the complete testing datasets of the BC. (DOCX) [file pone.0306073.s018.docx]

|  | **TiMiPNet**  **2D** | **TiMiPNet**  **3D** | **TiMiGNet**  **2D** | **TiMiGNet+ 2D** | **TiMiGNet**  **3D** |
| --- | --- | --- | --- | --- | --- |
|  | **PSNR** | | | | |
| **FULL** | 26.160186 | 23.470088 | 27.338041 | 27.419934 | 26.307368 |
| **FG** | 26.725306 | 24.092512 | 28.605868 | 28.653437 | 27.709028 |
| **BG** | 35.637719 | 32.672569 | 34.131415 | 34.264902 | 32.676855 |
|  | **SSIM** | | | | |
| **FULL** | 0.680092 | 0.749703 | 0.847917 | 0.854201 | 0.819653 |
| **FG** | 0.952000 | 0.935226 | 0.970061 | 0.970458 | 0.96874 |
| **BG** | 0.708751 | 0.787107 | 0.862053 | 0.868768 | 0.832051 |
|  | **MSE** | | | | |
| **FULL** | 0.002664 | 0.005256 | 0.002467 | 0.002421 | 0.002641 |
| **FG** | 0.002381 | 0.004689 | 0.00204 | 0.001987 | 0.002063 |
| **BG** | 0.000283 | 0.000566 | 0.000427 | 0.000435 | 0.000578 |
