# Supplementary material for: Virtual tissue microstructure reconstruction across species using generative deep learning
Source: PLoS One. 2024 Jul 12;19(7):e0306073. doi: 10.1371/journal.pone.0306073 (PMC11244806; doi:10.1371/journal.pone.0306073)
Supplement: Attachment — Submitted filename: ResponsetoReviewers.pdf [file pone.0306073.s019.pdf]

Universität
Konstanz

Hernán Morales-Navarrete

Universität Konstanz

Universitätsstraße 10, 78464 Konstanz

Email: hernan.morales-navarrete@uni-konstanz.de

Tel.: 07531 88 5547

Dr. Emily Chenette

Editor-in-Chief

PLOS ONE

26 May 2024

Dear Emily Chenette,

We sincerely appreciate the thoughtful and detailed feedback on our manuscript from you and the reviwers. We have carefully considered each of the comments and have made the necessary revisions to address them. Below is a point-by-point response to each of the reviewers' comments:

Reviewer #1:

1. What is the training and testing framework of TiMiPNet, TiMiGNet, and TiMiGNet+? Figure 1 only shows a training framework of TiMiGNet. What is the input and output of these networks?

Thank you for pointing this out. We have added a new figure (S1_Fig A) illustrating the training framework for TiMiPNet. Additionally, we have provided explanations on the differences in the training frameworks of TiMiGNet and TiMiGNet+ (lines 188 to 195). The input for the networks are 64x64 or 128x128 image patches of membranes, and the output for the networks are 64x64 or 128x128 image patches of bile canaliculi. Corresponding figures (S1_Fig B, C) showing the testing/prediction frameworks have also been included.

2. Lack of comparative experiments for predictor and generator. Please compare with other deep learning methods.

We agree that a comparison of several deep learning methods is needed and have included them. Specifically, we used Unet (TiMiPNet), CycleGAN (TiMiGNet), and added Pix2Pix to our analysis. Figures F4 Sup a,b demonstrate similar performance across architectures for predicting BC structures. We have expanded our specific case with CycleGAN by modifying

the loss function (TiMiGNet+), achieving better results for large structures like sinusoids (F3 Sup). We also evaluated the generator performance for CycleGAN-like architectures as suggested (F4 Sup c,d).

3. Please use a table to show the average PSNR and average SSIM on the complete testing dataset.

We have added a table displaying the average PSNR and SSIM for the complete testing dataset.

4. The pixels of the data picture are too low, and the text in some pictures can't be seen clearly.

We have exported all images in higher quality and increased the text size for clarity.

5. Please indicate the number of images used for training and testing.

We apologize for the lack of clarity. The number of images and patches used for training and testing are now described in the "Model Training" and "Quality Evaluation Metrics" sections of the Methods.

6. Please indicate the PSNR and SSIM under each sample in each figure.

We have added the average PSNR and SSIM values for each predicted image.

7. In short, the logic of the paper needs to be reorganized. There are some parameters that need to be explained in detail.

Thank you for the comment. We have reorganized the text for better logical flow and provided detailed explanations for relevant parameters.

Reviewer #2:

1. A quantitative evaluation was conducted, but quantitative numbers were not mentioned in the results section. Specific descriptions of these are needed.

Thank you for the comment. We clarified this by adding a summary table (S1 Table) of the average algorithm performance results and incorporated specific quantitative numbers into the results section.

2. Is it possible to compare the performance of the proposed model with the default CycleGAN?

We performed a detailed comparison as explained in our response to Reviewer #1, point 2.

3. What diseases can be better discovered using the proposed algorithm? The clinical significance needs to be further elucidated.

Thank you for raising this point. We have added a paragraph discussing the clinical significance and the potential diseases that can be better discovered using the proposed algorithm (lines 496 to 508).

4. There are disadvantages of GAN. The following paper introduces the shortcomings. "Application of generative adversarial networks (GAN) for ophthalmology image domains: a survey" Were there any experimental results like this? Discuss further the disadvantages.

Thank you for raising this point. We have addressed the disadvantages of GANs, as discussed in "Application of Generative Adversarial Networks (GAN) for Ophthalmology Image Domains: A Survey". We included a discussion on mode collapse, spatial deformity, unintended changes due to domain differences, and checkerboard artifacts in the final section of the manuscript (lines 510 to 521).

Reviewer #3:

The manuscript is well written and structured in a clear and logical way to present the new TiMiGNet solution. The authors discussed the methods, results, and debates thoroughly and thoughtfully. The conclusions are strongly supported by the comprehensive evidence. I like the idea of using TiMiGNet for biotissue image recognition and segmentation. The novel approach overcomes some of the existing challenges of biotissue image segmentation. I recommend the editor to accept this manuscript for publication.

One minor edit for authors is to replace all graphs with high-resolution images.

We appreciate your positive feedback and recommendation for publication. We have replaced all graphs with high-resolution images as suggested.

We hope these revisions adequately address all your comments and improve the quality and clarity of our manuscript. Thank you for your valuable feedback and consideration.

Sincerely yours,

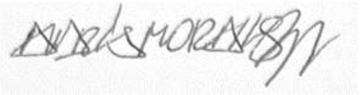

Hernán Andrés Morales-Navarrete
